# Cognitive Impairment and Depressive Symptoms Among Low-Income Hispanic and Non-Hispanic White Older Adults: A Cross-Sectional Analysis

**DOI:** 10.53520/rhm2026.104176

**Published:** 2026-01-02

**Authors:** Cecil Latta, Janet Lopez, Dahee Kim, Kworweinski Lafontant, Nicole Haberland, Trucvy Nguyen, Ayse Malatyali, Ladda Thiamwong

**Affiliations:** 1 Department of Psychology, University of Central Florida, Orlando, FL, USA; 2 College of Nursing, University of Central Florida, Orlando, FL, USA; 3 Institute of Exercise Physiology and Rehabilitation Science, University of Central Florida, Orlando, FL, USA; 4 College of Medicine, University of Central Florida, Orlando, Florida, USA; 5 Disability, Aging, and Technology Cluster, University of Central Florida, Orlando, FL, USA

**Keywords:** Mental health, Cognitive functioning, Minority populations, Aging

## Abstract

**Introduction:**

Hispanic older adults represent a rapidly growing demographic in the United States and may be disproportionately affected by cognitive impairment and depression. This study aimed to examine ethnic differences in the prevalence of cognitive impairment and depression among low-income Hispanic and non-Hispanic White older adults, and to explore the relationship between cognitive impairment and depressive symptoms within these groups.

**Methods:**

This cross-sectional study included 157 older adults (age = 76.3 ± 6.7 years, female = 133), recruited from independent living facilities and low-income community centers in Central Florida. The sample consisted of Hispanic (n = 91) and non-Hispanic White (n = 66) older adults. Cognitive impairment was assessed using the Memory Impairment Screen, and depression was measured using the Patient Health Questionnaire-9 (PHQ-9). Chi-square tests and Mann-Whitney U tests were used to analyze group differences.

**Results:**

A significant difference in cognitive impairment prevalence was found between ethnic groups (χ^2^ = 7.528, p = .006), with Hispanic participants showing higher rates. However, depression scores did not significantly differ between Hispanic and non-Hispanic White participants (U = 2930.50, p = .79), nor between those with and without cognitive impairment within each ethnic group (Hispanic: U = 839.00, p = .71; non-Hispanic White: U = 227.50, p = .93).

**Conclusions:**

Cognitive impairment is more prevalent among low-income Hispanic older adults compared to their non-Hispanic White counterparts. These findings emphasize the need for culturally tailored interventions to improve cognitive health care access for older adults.

## Introduction

Cognitive impairment and depression represent two of the significant health concerns facing aging populations, with particularly profound implications for Hispanic older adults.^1^ Currently, Hispanics account for approximately 9% of the United States (U.S.) older adult population (> 65 years old), and this percentage is projected to increase to 21% by 2060.^2^ Given this demographic shift, addressing the intersection of cognitive and mental health outcomes in this demographic is critical for the future of equitable mental health care in the U.S.^3^

Declining cognition can undermine older adults’ capacity to regulate mood, sustain meaningful social engagement, and employ coping strategies to counter depression.^4^ These issues are prominent in Hispanic older adults, who face disproportionate risks for cognitive impairment. Hispanics are nearly twice as likely as non-Hispanic Whites to develop cognitive impairment,^5^ and cultural norms may discourage disclosure of symptoms due to fears of burdening family members.^6^ This cultural norm may result in delayed diagnosis of cognitive impairment until it severely impacts older adults’ functioning, particularly given the centrality of family support in Hispanic cultural values.^7^

Recent evidence has highlighted racial and ethnic disparities in the progression of cognitive decline. Boza-Calvo et al.^8^ found that Hispanics had a significantly greater risk of progressing from subjective cognitive concerns (SCC) to mild cognitive impairment (MCI) over two years compared to non-Hispanic Whites. The findings suggest that Hispanic ethnicity may confer elevated vulnerability to cognitive decline, particularly when mood symptoms or other psychosocial stressors are present.^8^ Similarly, Persin et al.^9^ reported that depressive symptoms predicted cognitive functioning both cross-sectionally and longitudinally, with more potent effects observed among ethno-racial minority individuals than among non-Hispanic Whites.

Depression is also highly prevalent in the older Hispanic population, with estimates ranging from 13% to 35% of older Hispanic reporting significant depressive symptoms, depending on geographic region.^10^ This elevated psychological burden is partly explained by the high prevalence of chronic conditions such as diabetes and cardiovascular disease, which are strongly associated with depression and cognitive decline.^11,12^ Furthermore, Hispanic individuals with depression are less likely than their non-Hispanic Whites counterparts to access treatment due to barriers such as socioeconomic disadvantage, limited healthcare access, and language discordance hindering their quality of care.^3,13^

Notably, evidence indicates that cognitive impairment and depression are bidirectionally linked, each exacerbating the other.^14^ Cognitive impairment undermines functional independence, while depression accelerates the decline in cognition.^14^ Together, these two conditions perpetuate a downward spiral of mental and cognitive health.^15^ Comparative studies highlight that Hispanic older adults often enter late life with lower baseline cognitive scores and experience steeper age-related declines than their non-Hispanic White peers.^16^ Living in socioeconomically disadvantaged neighborhoods was associated with a decrease in performance across various cognitive domains, particularly among older Mexican American adults.^17^ Moreover, the reciprocal link between cognition and depression appears firmer among ethno-racial minority populations,^9^ where maybe cultural and structural barriers exacerbate the cycle of decline relative to non-Hispanic White. These findings underscore the urgent need for further research on the interplay of cognitive impairment and depression in Hispanic older adults. Thus, this study builds on prior research by examining how cognitive impairment and depression co-occur among low-income Hispanic older adults and the factors that may influence the cognitive impairment-depression relationship. The purpose of this study was to comprehensively evaluate the prevalence and the association between cognitive impairment and depression in Hispanic and non-Hispanic White older adults. We hypothesize that Hispanic older adults will have a higher prevalence of both conditions and that the association between cognitive impairment and depression will be stronger in this group.

## Scientific Methods

### Participants and Procedure

We analyzed the data of 157 older adults, comprising of 91 Hispanic (58.0%) and 66 non-Hispanic White (42.0%) individuals, who were engaged in a fall preventive intervention program as part of a larger cluster randomized controlled trial investigating the integration of technology-based physical and cognitive interventions to prevent falls among low-income older adults.^18^ The program was launched by the College of Nursing, University of Central Florida (UCF) after the approval of the UCF Institutional Review Board (IRB#: STUDY00003206). The program was also pre-registered on ClinicalTrials.gov (NCT05778604).

The program participants were recruited through senior apartment and community/neighborhood centers in low-income areas throughout the greater Orlando, Florida, USA metropolitan area via a partnership with the Mayor’s Committee on Livability and Healthy Aging. The eligibilities for the program were: older adults who (1) were aged 60 and above, (2) were classified as low-income in Orlando, Florida, as defined by the 2019 U.S. Census poverty thresholds based on family size and the number of children aged under 18 years,^19^ (3) reported a score of ≥ 22 on the Rowland Universal Dementia Assessment Scale: Multicultural Cognitive Assessment Scale (RUDAS),^20^ and (4) were able to stand on the balance plate without any assistance. Older adults were excluded from the program participation if they were: (1) living with medical conditions that contraindicate physical activity (e.g., shortness of breath), (2) currently receiving treatment at a rehabilitation facility, (3) planning to relocate within one year from date of recruitment, (4) hospitalized more than three times in the past 12 months due health conditions that limit the ability to safely engage in physical activity, and (5) unable to speak English or Spanish. Those who met the inclusion criteria were informed of the study’s purpose and procedures, provided with a detailed consent form, and given the opportunity to ask questions. Written informed consent was obtained from all participants prior to participation in the program. The program collected data from participants at four time-points: baseline (T1), eight weeks later (T2), three months later (T3), and six months later (T4) from the baseline. This study analyzed T1 data from participants who self-identified as Hispanic or non-Hispanic White and completed all measures of the research variables.

### Measurements

#### Cognitive Impairment.

Cognitive impairment was assessed using the Memory Impairment Screen (MIS).^21^ The MIS is a test of cognitive function and screening for Alzheimer’s disease among older adults. The MIS is a 4-minute-long assessment, consisting of a 4 item delayed free and cued recall memory test with controlled learning and high validity. The maximum score for the MIS is 8, and a score of 5–8 indicates no cognitive impairment, while a score equal to or less than 4 indicates possible cognitive impairment.^22,23^

#### Depressive Symptoms.

Depressive symptoms were assessed using the Patient Health Questionnaire-9 (PHQ-9).^24^ The PHQ-9 consists of nine items describing depressive symptoms (e.g., little interest or pleasure in doing things, feeling down, depressed, or hopeless) and participants reported how often they felt each symptom over the past two weeks on a four-point Likert scale (0 = not at all to 3 = nearly every day). The total score ranges from 0 to 27, with scores greater than 10 indicating moderate depression.^25^ Cronbach’s α of this measure was reported as 0.89 among older adults.^25,26^ In this study, Cronbach’s α of the PHQ-9 was 0.84.

#### Demographics and Health Variables.

Participants reported their age, sex, and race/ethnicity, education level (1 = less than high school, 2 = High school diploma, 3 = College and above), perceived financial stability (ranging from 1 = much less than adequate to 5 = much more than adequate), cohabitation status (1 = alone; 2 = partner/spouse; 3 = family/friends; 4 = others), and self-rated health (ranging from 1 = poor to 5 = excellent).

### Statistical Analysis

Descriptive and frequency analyses were performed to explore the characteristics of 157 participants. To investigate the group comparisons between Hispanic and non-Hispanic White older adults, independent samples t-test, chi-square test, and Mann-Whitney test were conducted after testing the normality of each research variable with Shapiro-Wilks. In this study, p ≤ 0.05 was considered statistically significant a priori, and SPSS 29.0 (IBM Corporation, Armonk, NY, USA)^27^ was utilized to analyze data. All data are presented as mean ± standard deviation unless otherwise indicated.

## Results

Table 1 presents demographic characteristics of the total participants and each racial/ethnic group. The total participants included 157 low-income older adults, of whom 58.0% identified as Hispanic (n = 91) and 42.0% as non-Hispanic White (n = 66). The mean age of the total participants was 76.30 years (SD = 6.66), with no statistically significant difference between Hispanic (76.36 ± 6.84 years old) and non-Hispanic White (76.22 ± 6.45 years old), t(155) = 0.137, p = .84. Participants were predominantly female (n = 133, 84.7%), and although a greater proportion of Hispanic was female (n = 81, 89.0%) compared to non-Hispanic White (n = 52, 78.8%), this difference was not statistically significant, χ^2^ (1) = 3.087, p = .08. Self-rated health was significantly lower among Hispanic (3.19 ± 0.84) than among non-Hispanic White (3.47 ± 0.86), U = 2409.00, p = .23. Educational attainment also differed significantly between groups, with a higher proportion of non-Hispanic White having completed college or above (n = 43, 65.2%) compared to Hispanic (n = 34, 37.4%), χ^2^ (2) = 16.141, p < .001. Likewise, perceived financial status varied significantly, χ^2^ (4) = 29.461, p < .001; with Hispanic more frequently reporting having “just enough“ or “less than adequate“ resources, while non-Hispanic White were more likely to report “more than adequate“ means. Finally, cohabitation status showed a statistically significant difference between groups, χ^2^ (3) = 11.427, p = .01. Hispanic were more likely to live alone (n= 62, 68.1%) than non-Hispanic White (n = 34, 51.5%), while non-Hispanic White were more likely to live with a partner or spouse (n = 26, 39.4%) compared to Hispanic (n = 15, 16.5%).

A chi-square test of independence was performed to examine the rates of cognitive impairment in both Hispanic and non-Hispanic White older adults. Table 2 presents the results of racial/ethnic group comparisons in cognitive function and depression. Out of the 157 participants, 36 (22.9%) met the criteria for cognitive impairment, while 121 (77.1%) did not. Among those with cognitive impairment, 28 (77.8%) were Hispanic, whereas only 8 (22.2%) were non-Hispanic White. In contrast, among the 121 participants without cognitive impairment, 63 (52.1%) were Hispanic, and 58 (47.9%) were non-Hispanic White. The chi-square test revealed a statistically significant difference in cognitive impairment rates between Hispanic and non-Hispanic White, χ^2^ (1) = 7.528, p = .006, suggesting that Hispanic older adults in this study were disproportionately affected by cognitive impairment.

We conducted Mann-Whitney U test to compare depression levels between Hispanic and non-Hispanic White older adults (See Table 2). The overall mean depressive symptom score for all participants was 3.9 ± 4.5, indicating little to no levels of depression symptoms. Hispanic had a mean depressive symptom score of 4.0 ± 4.8, while the non-Hispanic White had a mean depressive symptom score of 3.9 ± 4.3. The Mann- Whitney U test revealed no statistically significant difference in depression levels between the two ethnic groups, U = 2930.5, p = .79. These findings suggest that depression rates did not significantly differ between Hispanic and non-Hispanic White in this study.

A Mann-Whitney U test was conducted to assess whether cognitive impairment influenced depression levels within each ethnic group. Table 3 presents the cognitive group comparisons in depression of each racial/ethnic group. Across all participants, those with cognitive impairment (n = 36, 22.9%) had a mean depressive symptom score of 3.5 ± 4.2, while those without cognitive impairment (n = 121, 77.1%) had a mean score of 4.1 ± 4.7. The test revealed no statistically significant difference between these groups, U = 2090.5, p = .71.

When analyzed by race/ethnicity, the Hispanic cognitive impairment group (n = 28, 30.8%) had a mean depressive symptom score of 3.43 (SD = 4.17), while the Hispanic non-cognitive impairment group (n = 63, 69.2%) had a mean score of 4.25 (SD = 5.01) (See Table 3). However, the Mann-Whitney U test showed no significant difference in depression levels between those two groups, U = 839.00, p = .71. Similarly, for non-Hispanic White, those with cognitive impairment (n = 8, 12.1%) had a mean depressive symptom score of 3.9 ± 4.7, while those without cognitive impairment (n = 58, 87.9%) had a mean score of 3.8 ± 4.3, also showing no significant difference, U = 227.5, p = .93. These results indicate that while cognitive impairment rates were higher among Hispanic older adults, it was not significantly associated with higher depression scores in either ethnic group.

## Discussion

This study compared the prevalence of cognitive impairment, depression, and the relationship between cognitive impairment and depression between low-income Hispanic and non-Hispanic White older adults. Findings revealed that Hispanic participants had a significantly higher rate of cognitive impairment compared to their non-Hispanic White counterparts, suggesting that Hispanic older adults may be disproportionately affected by cognitive impairment. These results align with previous research indicating that Hispanic populations face a heightened risk for cognitive impairment and Alzheimer’s disease than their non-Hispanic White counterparts.^5,14,28^ Some contributing factors, such as lower educational attainment and reduced financial status, both of which were more prevalent among the Hispanic participants in this study, are known risk factors for cognitive impairment in older adults.^29^ Given the disparities in educational attainment and perceived financial status within our sample, the role of socioeconomic disadvantages in shaping cognitive impairment in Hispanic older adults cannot be overlooked and should be studied further. Clinically, the heightened risk of cognitive impairment among Hispanic older adults in low- income communities underscore the need for routine cognitive screening during annual physical exams using culturally sensitive tools. Interventions tailored to language, literacy levels, and cultural perceptions of memory loss are essential to improving outcomes in this population.

Interestingly, despite the differences in cognitive impairment between the two groups, no statistically significant differences were found in depression levels between Hispanic and non-Hispanic White low-income older adults or between the cognitively impaired and non- impaired individuals. Overall participants exhibited minimal depressive symptoms, as reflected by the low mean PHQ-9scores. Cognitive impairment was not associated with greater depression symptoms. This is different from other studies that usually find an association between depression and cognitive problems in older adults, suggesting a positive correlation between depression and cognitive impairment in older adults.^1,28,30^ These findings challenge assumptions of a uniform bidirectional association and highlight the need for culturally tailored strategies to address cognitive health in older Hispanic populations.

Several factors may explain these discrepancies. First, the overall low depression scores suggest that depressive symptoms were not prevalent in this cohort, potentially limiting the ability to detect group differences.

Second, it is possible that cultural factors^31^ and/or stigmas regarding mental health,^32,33^ may have influenced the reporting of depressive symptoms among Hispanic individuals. Mental illness is often stigmatized by Hispanic communities, where it associated with linked to personal weakness, the idea of ‘going crazy’, supernatural causes like witchcraft or demonic influence, or a lack of faith in God.^34–37^ Furthermore, Hispanic individuals often express depressive symptoms through physical complaints, which may affect the accuracy of PHQ-9 self-reports and contribute to misdiagnosis or delayed diagnosis.^38^ Although the PHQ-9 is widely used, it cultural and linguistic relevance to Hispanic population may be limited. Differences in the interpretation of depression-related questions on assessments like the PHQ-9 may influence the reporting of depressive symptoms in Hispanic populations.^10,39^ For example, the Spanish term ‘deprimido’ (depressed) may not align with how Hispanic individuals conceptualize emotional distress. Therefore, even though depression did not significantly correlate with cognitive impairment in this study, mental health services should remain a priority for this population, as depressive symptoms may go underreported in minority aging populations due to disparities in mental health screening and the high prevalence of unmet mental health needs, which can lead to underdiagnosis.^7,32^ A more reliable screening option may be the Spanish version of the CES-D, which has demonstrated higher validity than other Spanish-translated assessments, with a Cronbach’s α of 0.93.^40^

The Hispanic Health Paradox (HHP) describes how Hispanic living the U.S. often experience equal or better health outcomes compared to non-Hispanic Whites despite socioeconomic disadvantages, attributed to strong social support and cultural protective factors.^41^ Hispanics, especially immigrants, often show lower rates of depression than non-Hispanic Whites, demonstrating resilience despite socioeconomic disadvantages.^42^ Similarly, some Hispanic subgroups, particularly recent immigrants, tend to maintain better cognitive function or experience slower decline compared to non-Hispanic Whites.^43^ Although cognitive impairment differed between groups in our study, depression levels did not, may suggest that cultural resilience may protect against depression in Hispanic population experiencing cognitive aging. This insight supports tailoring interventions that leverage social and cultural strengths to enhance mental health outcomes among Hispanic older adults. Future research should investigate how the HHP and associated cultural, social, and environmental factors contribute to cognitive health and mental health outcomes among Hispanic older adults.

The lack of significant findings regarding depression and cognitive impairment in this study could be attributed to the small sample size of cognitively impaired participants, which could have limited statistical power, as multiple population-based studies reported positive relationship between depressive symptoms and cognitive impairment.

### Limitations

Several limitations should be acknowledged in this study. First, while cross-sectional designs are valuable for comparing two groups at one point in time, they limit the ability to draw conclusions about causality or changes in cognitive and emotional health over a longer period. Participants were recruited from a community-based fall prevention program and introducing selection bias, limiting generality. Additionally, participants were required to score ≥22 RUDAS scale, those with more serve cognitive impairment were likely underrepresented. This may have limited the variability in cognitive impairment and depression although our analyses used the Memory Impairment Screen (MIS). The relatively small number of participants with depression may have reduced the statistical power to detect meaningful differences in depression levels between cognitive impairment groups. Lastly, there are many barriers when it comes to recruiting the Hispanic population for research. Language barriers, transportation, negative stigma when it comes to mental health, and lack of English proficiency all play major roles in this barrier.^10^

## Conclusions

This study highlights a significant disparity in cognitive impairment among older adults, with Hispanic individuals in low-income communities experiencing higher rates than their non-Hispanic White counterparts. Socioeconomic disadvantages likely contribute to this gap. While depression levels did not differ significantly across groups, cultural factors may influence how symptoms are reported and detected. These findings underscore the need for culturally sensitive, accessible mental and cognitive health care tailored to underserved Hispanic populations. Clinical practice should prioritize routine cognitive screening during annual physical examinations using linguistically and culturally appropriate tools. Interventions that address language, literacy, and cultural perceptions of memory loss are critical to improving outcomes. Furthermore, integrated care models that combine cognitive and mental health screening and services with community-based resources may help mitigate disparities. Future research should include longitudinal designs that are recommended to track changes over time and determine the causal relation between cognitive impairment and depression. Additionally, increasing sample size and diversity will enhance representativeness and allow for more robust subgroup analyses.

## Figures and Tables

**Table 1.** Demographic and Health Characteristics of Hispanic and Non-Hispanic White Older Adults.

	Total Sample (N = 157)	Hispanic (n = 91, 58.0%)	Non-Hispanic White (n = 66, 42.0%)	p-value
	Mean *±SD/n(%)*	Mean *±SD/n(%)*	Mean *±SD/n(%)*	
Age	76.3 ± 6.7	76.4 ± 6.8	76.2 ± 6.5	.84
Sex				.08
Male	24 (15.3%)	10 (11.0%)	14 (21.2%)	
Female	133 (84.7%)	81 (89.0%)	52 (78.8%)	
Perceived Financial Status				< .001
Much Less Than Adequate	8 (5.1%)	7 (7.7%)	1 (1.5%)	
Less Than Adequate	22 (14.0%)	15 (16.5%)	7 (10.6%)	
Just Enough	96 (61.1%)	64 (70.3%)	32 (48.5%)	
More Than Adequate	28 (17.8%)	4 (4.4%)	24 (36.4%)	
Much More Than Adequate	3 (1.9%)	1 (1.1%)	2 (3.0%)	
Education Attainment				< .001
Less than high school	21 (13.4%)	19 (20.9%)	2 (3.0%)	
High school diploma	59 (37.6%)	38 (41.8%)	21 (31.8%)	
College or above	77 (49.0%)	34 (37.4%)	43 (65.2%)	
Cohabitation status				.01
Alone	96 (61.1%)	62 (68.1%)	34 (51.5%)	
Partner/Spouse	41 (26.1%)	15 (16.5%)	26 (39.4%)	
Family/Friends	16 (10.2%)	12 (13.2%)	4 (6.1%)	
Others	4 (2.5%)	2 (2.2%)	2 (3.0%)	
Self-rated health	3.3 ± 0.8	3.2 ± 0.8	3.5 ± 0.9	.02

Note. P-values are from non-parametric independent samples t-test (i.e., Mann-Whitney U tests) or crosstab analyses (i.e., Chi-square tests) between Hispanic and Non-Hispanic White.

**Table 2.** Comparison of Cognitive Function and Depression Between Hispanic and Non-Hispanic White Older Adults (N = 157).

	Total Sample (N=157)	Hispanic (n=91, 58.0%)	Non-Hispanic White (n=66, 42.0%)	
	*M±SD/n(%)*	*M±SD/n(%)*	*M±SD/n(%)*	*p*
Cognitive function				.006
Cognitive impairment	36 (22.9%)	28 (77.8%)	8 (22.2%)	
No cognitive impairment	121 (77.1%)	63 (52.1%)	58 (47.9%)	
Depression	3.9 ± 4.5	4.0 ± 4.8	3.9 ± 4.3	.79

Note. P-values are from non-parametric independent samples t-test or crosstab analyses.

**Table 3.** Depression by Cognitive Function Status in Hispanic and Non-Hispanic Older Adults (N = 157).

	Depression	
	*M±SD*	*p*
**Total Sample (N = 157)**	3.9 ± 4.5	
Cognitive function		
Cognitive impairment (n = 36, 22.9%)	3.5 ± 4.2	.71
No cognitive impairment (n = 121, 77.1%)	4.1 ± 4.7	
**Hispanic (n = 91)**		
Cognitive impairment (n = 28, 30.8%)	3.4 ± 4.2	.71
No cognitive impairment (n = 63, 69.2%)	4.3 ± 5.0	
**Non-Hispanic (n = 66)**		
Cognitive impairment (n = 8, 12.1%)	3.9 ± 4.7	.93
No cognitive impairment (n = 58, 87.9%)	3.8 ± 4.3	

Note. P-values are from non-parametric independent samples t-test.
